# Robust response to pembrolizumab in temozolomide-associated hypermutated and microsatellite instability-high functional pancreatic neuroendocrine tumor

**DOI:** 10.1093/oncolo/oyag229

**Published:** 2026-06-15

**Authors:** Udhayvir S Grewal, Charles W Shi, Saima Muzahir, Po H Ear, Seth J Concors, Daniel M Halperin

**Affiliations:** Department of Hematology and Medical Oncology, Winship Cancer Institute of Emory University, Atlanta, GA 30308, United States; Department of Internal Medicine, Emory University School of Medicine, Atlanta, GA 30308, United States; Department of Radiology and Imaging Sciences, Emory University School of Medicine, Atlanta, GA 30308, United States; Department of Hematology and Medical Oncology, Winship Cancer Institute of Emory University, Atlanta, GA 30308, United States; Division of Surgical Oncology, Winship Cancer Institute of Emory University, Atlanta, GA 30308, United States; Department of Hematology and Medical Oncology, Winship Cancer Institute of Emory University, Atlanta, GA 30308, United States

## Abstract

Pancreatic neuroendocrine tumors (PanNETs) are typically characterized by low tumor mutational burden and limited responsiveness to immune checkpoint inhibitors. Emerging evidence suggests that prior exposure to alkylating chemotherapeutic agents may be associated with a hypermutated phenotype (along with DNA mismatch repair dysfunction or DNA damage response gene alterations), potentially sensitizing tumors to immunotherapy. We present a case of a 68-year-old woman with metastatic functional PanNET (VIPoma) who developed a treatment-associated hypermutated, microsatellite instability-high phenotype following capecitabine-temozolomide therapy. Treatment with pembrolizumab resulted in a robust clinical, biochemical, and radiographic response. This case highlights dynamic genomic evolution in PanNETs and underscores the importance of serial molecular profiling in guiding therapeutic decisions.

Key Points

- Pancreatic neuroendocrine tumors (PanNETs) are typically immunologically “cold” tumors with tumor mutational burden (TMB)^low^ and limited response to immune checkpoint inhibitors.
- Alkylating chemotherapy may induce TMB^high^ and a microsatellite instability-high (MSI^high^) phenotype via DNA damage and mismatch repair alterations.
- Liquid biopsy represents a powerful tool that can capture dynamic genomic changes with therapeutic relevance.
- Hypermutated MSI^high^ PanNETs may demonstrate meaningful responses to immune checkpoint inhibition.

## Patient story

A 68-year-old woman with no significant past medical history was evaluated for progressive metastatic pancreatic neuroendocrine tumor (PanNET), WHO grade 3 (Ki-67 22.5%). She initially presented in the fall of 2022 with a 3.8 × 2.5 cm pancreatic head mass abutting the superior mesenteric artery and involving the superior mesenteric vein in the setting of abdominal pain and diarrhea. Biopsy confirmed a well-differentiated NET (Ki-67 22.5%). Immunohistochemistry showed tumor cells positive for CAM5.2 and synaptophysin, with patchy INSM1 positivity. Additional stains demonstrated DAXX with aberrant cytoplasmic expression, ATRX with retained nuclear expression, p53 with variable (wild-type pattern) staining, and SMAD4 with retained expression; Rb staining was equivocal. The patient received neoadjuvant capecitabine and temozolomide (CAPTEM) from October 2022 to February 2023 with a partial radiographic response and improvement in clinical symptoms. In April 2023, she underwent surgical resection during which a Whipple procedure was attempted but her pancreatic tumor was left in place due to major vessel involvement. She remained without evidence of disease progression on surveillance imaging until early 2025.

**Figure 1:**
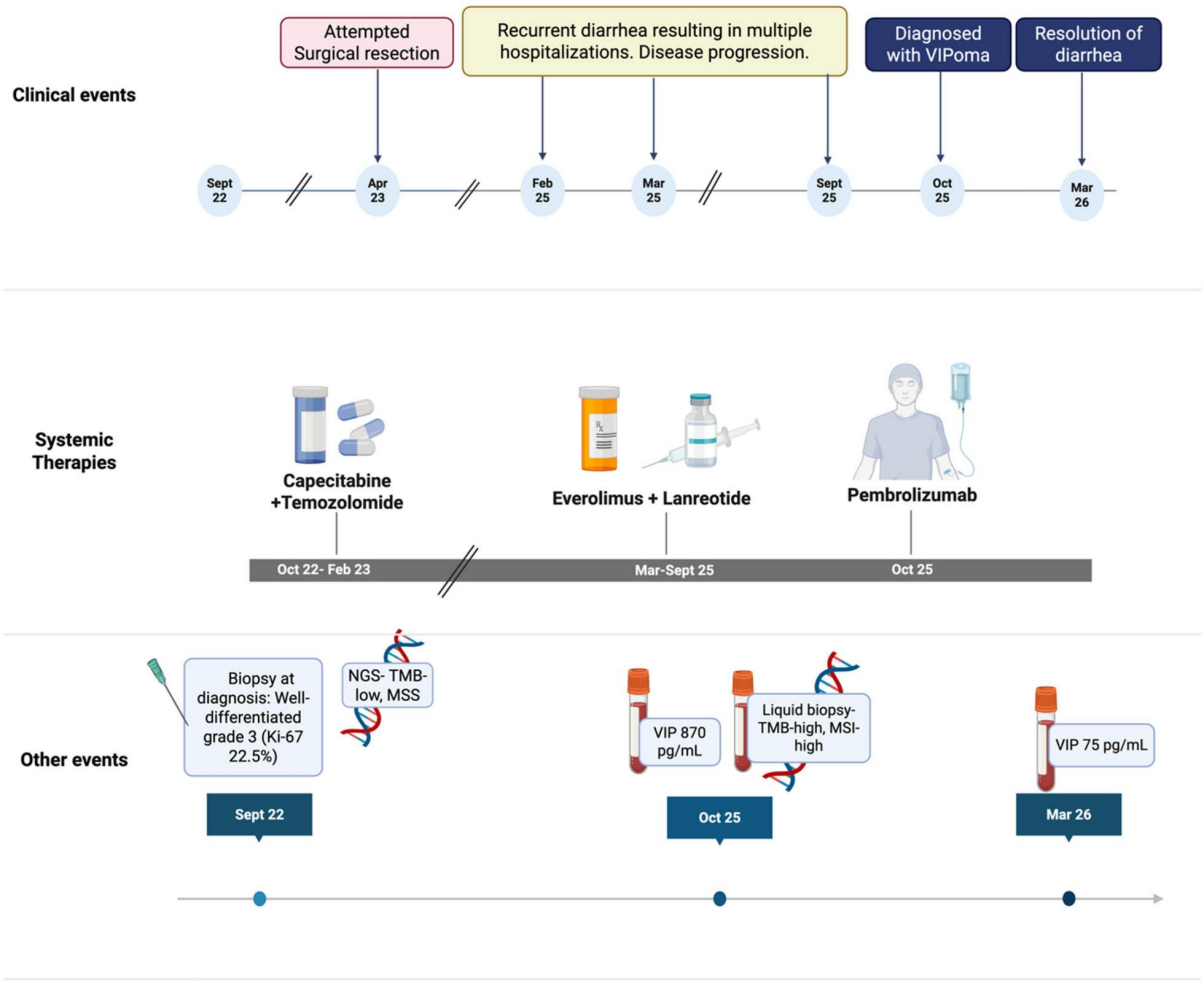
Clinical timeline and treatment course. This timeline illustrates the clinical course of a 68-year-old woman with well-differentiated grade 3 pancreatic neuroendocrine tumor (PanNET). Following initial diagnosis in September 2022 (Ki-67 22.5%), the patient received capecitabine and temozolomide from October 2022 to February 2023, followed by attempted surgical resection in April 2023. She remained under surveillance until early 2025, when recurrent disease was identified in the setting of worsening diarrhea and multiple hospitalizations. Systemic therapy with everolimus and lanreotide was administered from March to September 2025, with continued symptomatic progression. In October 2025, she was diagnosed with vasoactive intestinal peptide–secreting tumor (VIPoma), with markedly elevated serum vasoactive intestinal peptide (VIP) levels (870 pg/mL), and liquid biopsy demonstrated high tumor mutational burden (TMB^high^) and microsatellite instability–high (MSI^high^) status. Pembrolizumab was initiated in October 2025, resulting in rapid clinical improvement, normalization of biochemical abnormalities, and radiographic response, with resolution of diarrhea and reduction in VIP levels to 75 pg/mL by March 2026.

In early 2025, she noted worsening abdominal pain and diarrhea which was investigated with CT which demonstrated disease progression in the form of new hepatic metastatic disease and increase in the size of the pancreatic head mass. She was subsequently initiated on lanreotide and everolimus in March 2025, which were continued through September 2025 with persistent symptoms (diarrhea and abdominal pain). During this period, she required multiple hospitalizations for dehydration and diarrhea, the latter attributed to infectious (*Clostridium difficile* infection) and treatment-related (everolimus) etiologies. Despite therapy with fidaxomicin, oral vancomycin, telotristat, bile acid sequestrants, antidiarrheals and pancreatic enzyme replacement therapy, her diarrhea continued to worsen progressively. During a hospitalization in September 2025, laboratory evaluation revealed severe electrolyte derangements, including hypokalemia (2.6 mmol/L; reference range: 3.5–5.0 mmol/L), hyperchloremic metabolic acidosis (bicarbonate 10 mmol/L; reference range: 22–29 mmol/L, chloride 119 mmol/L; reference range: 98–106 mmol/L), with preserved renal function, consistent with ongoing gastrointestinal losses. Figure 1 shows the clinical timeline and treatment course.

**Figure 2:**
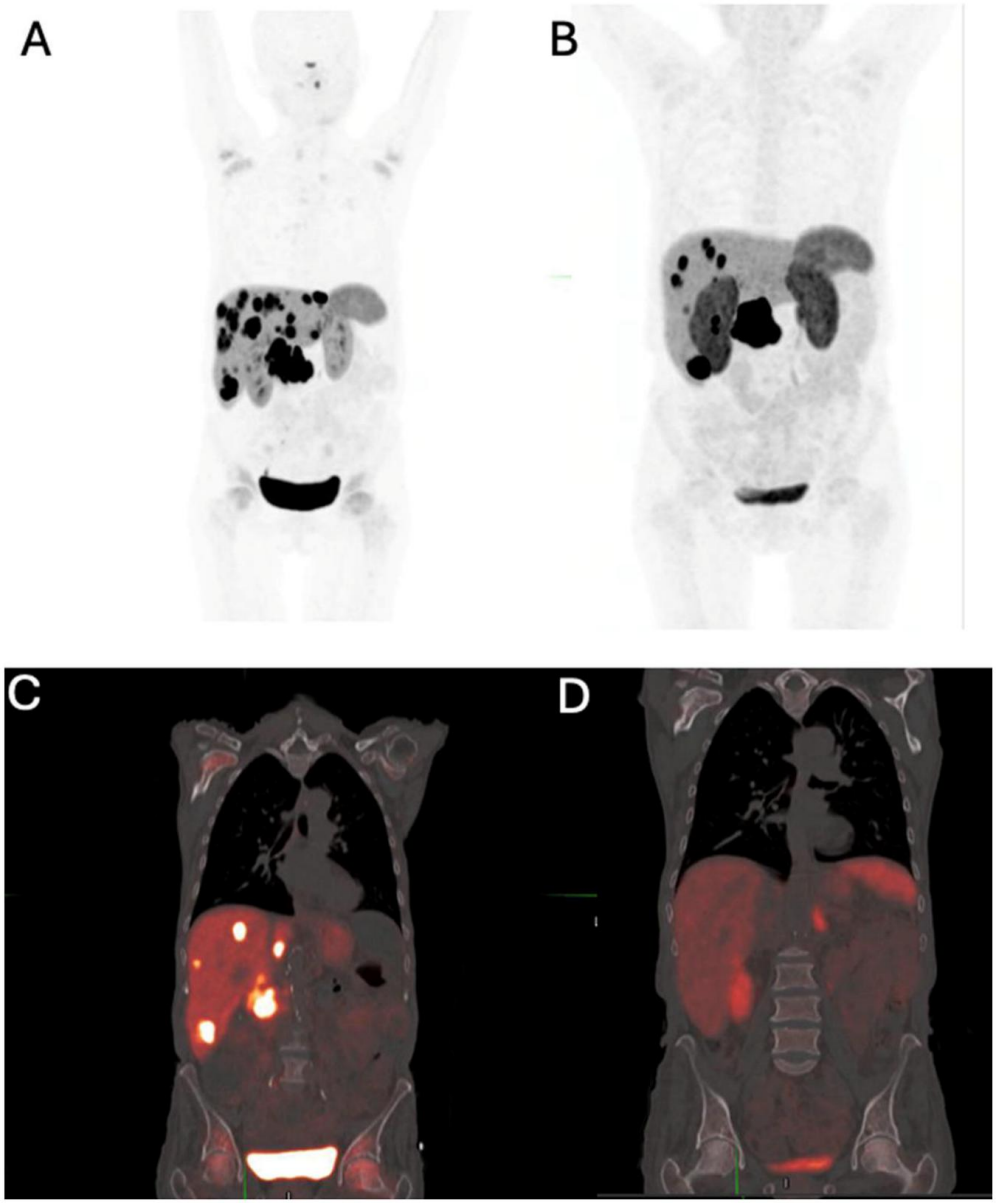
Radiographic response to pembrolizumab on ^64^Cu-DOTATATE PET/CT imaging. Maximum intensity projection (MIP) images (A, B) and corresponding fused PET/CT images (C, D) are shown. Baseline imaging obtained in October 2025 (A, C) demonstrates extensive somatostatin receptor-avid metastatic disease involving the liver and abdominal cavity. Follow-up imaging after four cycles of pembrolizumab in March 2026 (B, D) shows a marked reduction in radiotracer uptake and overall tumor burden, consistent with a significant treatment response.

She was subsequently discharged and referred to our institution in October 2025 for evaluation. Given refractory high-volume diarrhea, electrolyte abnormalities, and metastatic disease, a functional syndrome associated with PanNET was suspected. Biochemical testing demonstrated markedly elevated vasoactive intestinal peptide (VIP) levels (870 pg/mL, reference range <86 pg/mL), confirming VIPoma syndrome. Repeat imaging with ^64^Cu-DOTATATE positron emission tomography (PET) imaging confirmed pancreatic and multifocal bilobar hepatic disease, and biopsy confirmed well-differentiated NET (Figure 2A, C).

## Molecular tumor board

### Genotyping results and interpretation of the molecular results

Initial tissue-based next-generation sequencing (Caris Life Sciences^®^, October 2022) demonstrated low tumor mutational burden (5 mut/Mb), microsatellite stability, low genomic loss of heterozygosity (1%), and pathogenic alterations in ARID1A (p.E1683*), *TP53 (p.R273C)*, and FAT1 (p.K2537*), along with variants of uncertain significance in *ATM* and *NTRK1*. At disease progression in October 2025, liquid biopsy (Guardant360^®^) revealed a markedly altered genomic landscape characterized by a substantially elevated TMB (193.38 mut/Mb), microsatellite instability-high (MSI^high^) status, and pathogenic somatic alterations including ARID1A E1683* (2.2%), *SMARCA4 R1157W* (1.8%), *SETD2 T1652Yfs*14 (1.5%)*, MET G1087E* (0.4%)*, MSH6 R240* (0.2%), and *TP53 C242Y* (0.05%). The assay has been validated in the past for the assessment of MSI^high^ phenotype across solid tumors with methodology detailed in a prior publication.^1^ A liquid biopsy was performed at progression instead of repeat tissue biopsy per patient preference. Collectively, these findings demonstrate a clear transition from a genomically stable tumor to a TMB^high^, MSI^high^ phenotype, consistent with genomic evolution associated with the acquisition of mismatch repair (MMR) deficiency.

### Functional and clinical significance of the specific mutation in the particular cancer

**Figure 3:**
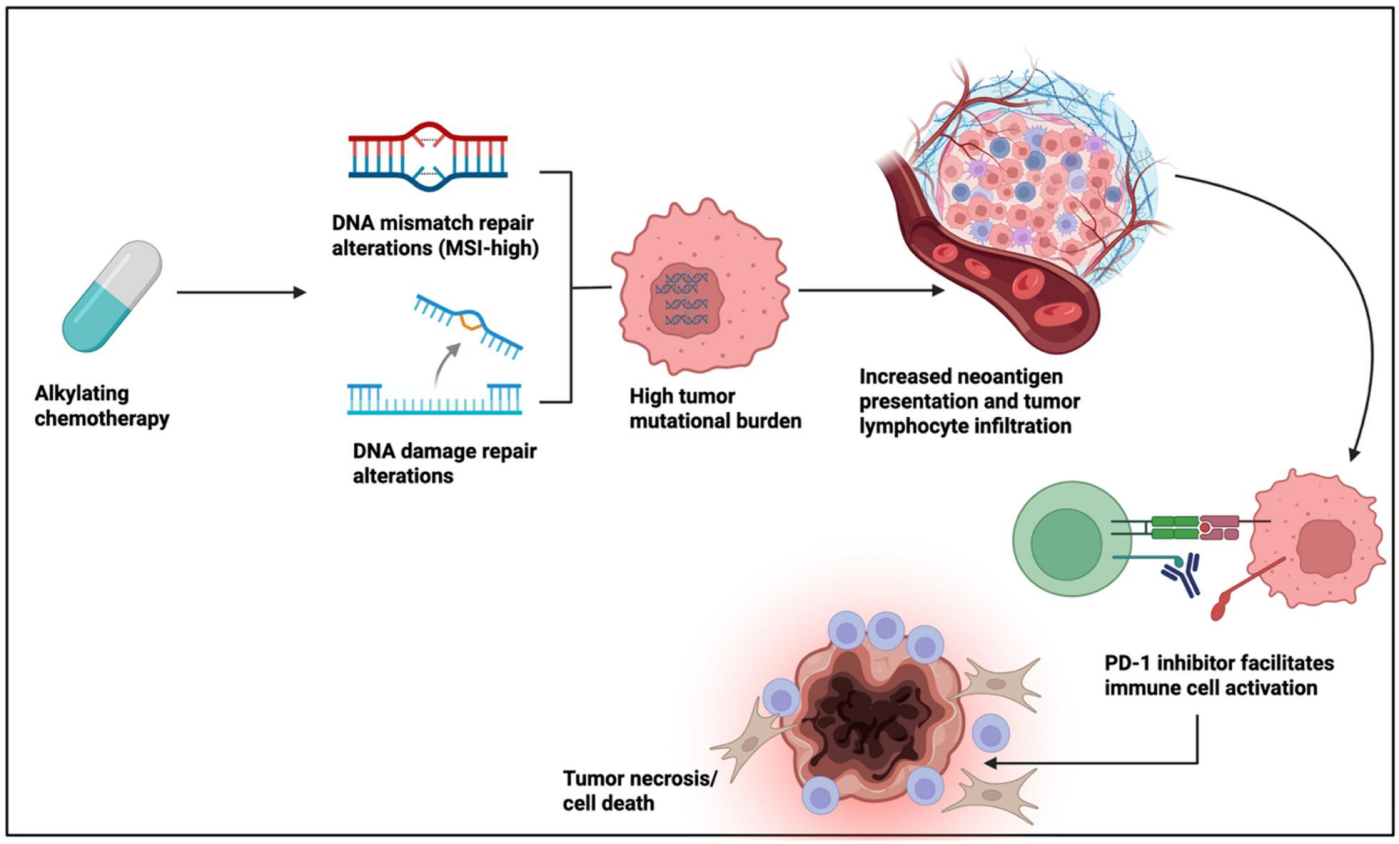
Proposed mechanism of alkylating chemotherapy-associated hypermutation and enhanced sensitivity to immune checkpoint inhibition in pancreatic neuroendocrine tumors. Exposure to alkylating chemotherapy (e.g., temozolomide) induces DNA damage, leading to alterations in DNA damage repair pathways and, in select cases, dysfunction of the mismatch repair system. This results in the emergence of a hypermutated phenotype characterized by high tumor mutational burden (TMB) and microsatellite instability–high (MSI-H) status. The increased mutational load promotes neoantigen generation and enhanced antigen presentation, facilitating tumor-infiltrating lymphocyte recruitment and an inflamed tumor microenvironment. Immune evasion through programmed cell death protein 1 (PD-1) signaling can be overcome with PD-1 inhibitors (e.g., pembrolizumab), leading to T-cell activation, tumor cell recognition, and cytotoxic tumor cell death.

Following temozolomide exposure, the patient’s WHO grade 3 PanNET evolved from TMB^low^ (5 mut/Mb) to TMB^high^ (193.38 mut/Mb) with an acquired MMR (*MSH6*) mutation and an MSI^high^ phenotype. This observation underscores that treatment-associated genomic evolution can drive a TMB^high^/MSI^high^ phenotype, highlighting a distinct biological pathway that may potentially confer meaningful sensitivity to immune checkpoint inhibitors (ICI) (Figure 3). While traditionally considered to be immunologically “cold” tumors, prior data show a strong signal of activity in a subset of patients with acquired TMB^high^ tumors. In a cohort of 64 patients with heavily pretreated PanNETs (median Ki-67 28%), TMB^high^ tumors were frequent (73%), with MMR deficiency on immunohistochemistry (IHC) present in 39% of tumors. Notably, TMB^high^ tumors had greater prior alkylating chemotherapy exposure (median 15 vs 10 cycles; second alkylator 48.7% vs 11.1%; *P* = .003) and more frequent peptide receptor radionuclide therapy (PRRT) use (64.9% vs 40.7%), supporting a treatment-associated hypermutation phenotype.^2^ In another multinational prospective cohort, ∼31% of patients developed a TMB^high^ phenotype following progression on alkylating chemotherapy, with the median TMB exceeding 50 mut/Mb. This hypermutation appeared to be treatment-related, associated with longer exposure to CAPTEM and enriched in patients previously treated with PRRT, suggesting cumulative DNA damage as a driver.^3^ Clinically, this genomic shift may also be seen as a co-occurrence to high-grade progression, wherein a WHO Grade 1/2 tumor may evolve into Grade 3 disease, the latter usually associated with a more aggressive disease biology.^4^ In the current case however, the patient had *de novo* WHO Grade 3 PanNET and there was no clinical concern for high-grade progression or transformation.

### Potential strategies to target the pathway and implications for clinical practice

PanNETs account for approximately 1%–2% of pancreatic neoplasms and represent a biologically and clinically heterogeneous disease group.^5^ Although most PanNETs are nonfunctional, a minority are characterized by clinical syndromes associated with an overproduction of bioactive peptides. VIP-omas are among the rarest functional NETs and are characterized by profuse watery diarrhea, hypokalemia, and metabolic acidosis associated with an abnormal overproduction of VIP.^6^ While a host of different therapeutic options are now available for management of advanced PanNETs, immune checkpoint inhibitors (ICIs) have had rather limited therapeutic success in the treatment of patients with advanced disease. This is primarily attributed to the relatively cold tumor immune microenvironment owing to a paucity of tumor infiltrating lymphocytes and a low TMB, generally ranging from 0.1 to 5 mutations per megabase. However, emerging data suggest that prior treatment with alkylating chemotherapy, such as temozolomide, may induce a TMB^high^ phenotype in a subset of PanNETs through MMR dysfunction and/or DNA damage repair (DDR) pathway alterations.^7^^,^^8^ This phenomenon has garnered particular interest as these changes may render an exquisite therapeutic vulnerability to ICIs, although clinical data related to outcomes remain largely heterogeneous.^2^^,^^4^^,^^9^^,^^10^

Across datasets, the efficacy of ICIs in this setting appears modest overall but enriched in subsets with acquired MSI^high^ disease. In the previously described prospective cohort study (*n* = 32), among patients treated with ICIs, outcomes ranged from primary progressive disease to durable radiographic responses. Among 5 patients with TMB^high^ disease treated with ICIs, 40% (2/5) had primary progressive disease, 20% (1/5) achieved transient disease control (∼8 months), and 40% (2/5) had major ongoing responses, with an overall clinical benefit rate of 60%. Notably, the most pronounced benefit was observed in 2 patients harboring pathogenic MMR and *MUTYH* alterations, suggesting that TMB alone is insufficient as a predictive biomarker.^3^ This signal is reinforced by the international retrospective study by de Mestier et al. which reported an overall response rate of 17% and median progression-free survival (PFS) of 3.2 months in heavily pretreated but TMB-unselected PanNETs. However, patients with TMB^high^ tumors had improved responses (overall response rate, ORR 30% vs 0%), and those with MMR deficiency derived the greatest benefit (ORR 44%, median PFS 8.9 months). Multivariable analyses confirmed MMR deficiency as an independent predictor of response, underscoring the importance of integrating genomic context beyond TMB alone.^2^ In addition to these studies, anecdotal evidence from individual case reports also highlights the therapeutic benefit of ICIs in patients with NENs and acquired TMB^high^ disease, mostly in the setting of MSI^high^ phenotype.^9^^,^^11^

Despite these encouraging signals, substantial heterogeneity in outcomes with ICIs in an unselected cohort of TMB^high^ PanNETs should be acknowledged. Responses in clinical practice may be rather inconsistent, likely due to spatiotemporal genomic heterogeneity which is a hallmark feature of NETs including PanNETs.^12^ Moreover, other malignancies that have traditionally been considered “immunologically cold,” such as microsatellite stable colorectal cancer, are also known to acquire a TMB^high^ phenotype after exposure to targeted therapy. However, this apparent genomic shift does not render these tumors therapeutically vulnerable to ICI as the rise in TMB is thought to occur due to subclonal alterations unique to individual disease sites that are inadequate to elicit a robust antitumor immune response.^13^ Furthermore, similar observations in other malignancies such as glioblastoma suggest that alkylating chemotherapy-associated hypermutation does not uniformly predict immunotherapy benefit.^14^

## Patient update

Given the tumor-agnostic approval for MSI^high^ malignancies, pembrolizumab was initiated in October 2025. The patient was not on any concurrent therapies at the time of initiation of pembrolizumab therapy. Four weeks after initiation of treatment, the patient noted significant improvement in her diarrhea and started regaining weight. At her follow-up visit following four cycles of pembrolizumab (March 2026), the patient was noted to have marked clinical improvement with near-complete resolution of diarrhea accompanied by continued gain of lost weight and improvement in her performance status. Notably, she reported no recurrent hospitalizations; electrolyte abnormalities normalized, with potassium improving to 4.0 mmol/L (reference range: 3.5–5.0 mmol/L) and magnesium to 2.0 mg/dL (reference range: 1.7–2.2 mg/dL), along with resolution of metabolic acidosis. Repeat VIP levels normalized to 75 pg/mL (reference range: <86 pg/mL). Follow-up ^64^Cu-DOTATATE PET/CT in March 2026 demonstrated a decrease in the number of hepatic metastases with resolution of left hepatic lesions and relative stability of the mesenteric mass with no new metastatic lesions identified (Figure 2B–D). The patient remains on therapy with pembrolizumab.

Overall, our case further highlights that the use of ICI in acquired TMB^high^/MSI^high^ PanNET is feasible even in the setting of functional disease (VIPoma) and can yield a robust multimodal response. Additionally, we show that liquid biopsy may serve as a powerful, noninvasive tool to identify patients harboring tumors with an acquired TMB^high^/MSI^high^ phenotype. Our case also supports the growing interest in leveraging individual molecular portraits of each tumor for personalizing therapeutic decision-making for patients with NETs.^15^ However, prospective clinical studies with integrated translational work are needed to better identify predictive biomarkers associated with meaningful responses to ICIs in this patient population.

## Data Availability

Additional clarifications/data pertaining to the case are available upon request.

## References

[1] Willis J , Lefterova MI, Artyomenko A, et al Validation of microsatellite instability detection using a comprehensive Plasma-Based genotyping panel. Clin Cancer Res. 2019;25:7035-7045. 31383735 10.1158/1078-0432.CCR-19-1324

[2] de Mestier L , Halfdanarson TR, Apostolidis L, et al Immunotherapy for metastatic pancreatic neuroendocrine tumors with high mutational burden and mismatch repair alterations following treatment with alkylating chemotherapy. Endocr Pathol. 2025;36:42. 10.1007/s12022-025-09887-8 41182464

[3] Trevisani E , Cingarlini S, Gomes Taboada R, et al Prospective multinational evaluation of alkylating-induced hypermutation in neuroendocrine neoplasms (NEN): clinical and molecular profiles associated with response to immune checkpoint inhibitors (CPI). Ann Oncol. 2024;35:S755.

[4] de Mestier L , Cohen D, de Rycke O, et al Temozolomide treatment induces an MMR-dependent hypermutator phenotype in well-differentiated pancreatic neuroendocrine tumors. Abstract #4194. Presented at: 21st Annual ENETS Conference 2024; 2024.

[5] Young K , Iyer R, Morganstein D, Chau I, Cunningham D, Starling N. Pancreatic neuroendocrine tumors: a review. Future Oncol. 2015;11:853-864. 25757686 10.2217/fon.14.285

[6] Hofland J , Falconi M, Christ E, et al European neuroendocrine tumor society 2023 guidance paper for functioning pancreatic neuroendocrine tumour syndromes. J Neuroendocrinol. 2023;35:e13318. 37578384 10.1111/jne.13318

[7] Taboada RG , Torrezan GT, Riechelmann RP. Alkylating-induced hypermutation in pancreatic neuroendocrine tumours. BMJ Oncol. 2025;4:e000814. 10.1136/bmjonc-2025-000814 PMC12612724 41244370

[8] Hackeng WM , Dreijerink KM, Brosens LA. Should we worry about high-grade pancreatic neuroendocrine tumor progression and alkylating agents?. J Pathol. 2025;266:1-4. 40007046 10.1002/path.6409

[9] Klempner SJ , Hendifar A, Waters KM, et al Exploiting Temozolomide-Induced hypermutation with pembrolizumab in a refractory High-Grade neuroendocrine neoplasm: a proof-of-concept case. JCO Precis Oncol. 2020;4:614-619. 35050748 10.1200/PO.20.00034

[10] de Mestier du Bourg L , Cohen D, Masliah-Planchon J, et al Temozolomide treatment induces an MMR-dependent hypermutator phenotype in well differentiated pancreatic neuroendocrine tumors. Ann Oncol. 2023;34:S701.

[11] Sun F , Grenert JP, Tan L, et al Checkpoint inhibitor immunotherapy to treat Temozolomide-Associated hypermutation in advanced atypical carcinoid tumor of the lung. JCO Precis Oncol. 2022;6:e2200009. 35737914 10.1200/PO.22.00009 PMC9249272

[12] Lou X , Qin Y, Xu X, Yu X, Ji S. Spatiotemporal heterogeneity and clinical challenge of pancreatic neuroendocrine tumors. Biochim Biophys Acta Rev Cancer. 2022;1877:188782. 36028148 10.1016/j.bbcan.2022.188782

[13] Yeh C , Artz O, Zhang H, et al Acquired high tumor mutational burden and activity of immunotherapy after targeted therapy in microsatellite stable colorectal cancer. Clin Cancer Res. 2026;32:1100-1109. 41165465 10.1158/1078-0432.CCR-25-2566 PMC13012239

[14] Ahmad H , Fadul CE, Schiff D, Purow B. Checkpoint inhibitor failure in hypermutated and mismatch repair-mutated recurrent high-grade gliomas. Neurooncol Pract. 2019;6:424-427. 31832212 10.1093/nop/npz016 PMC6899050

[15] Grewal US , Clarke CN, Kurzrock R. Neuroendocrine neoplasm classification: the missing piece of the puzzle. Med. 2025;6:100897. 41240896 10.1016/j.medj.2025.100897

